# Adjusting growth standards for fetal sex improves correlation of small babies with stillbirth and adverse perinatal outcomes: A state-wide population study

**DOI:** 10.1371/journal.pone.0274521

**Published:** 2022-10-10

**Authors:** Natasha L. Pritchard, Susan P. Walker, Alexandra R. Mitchell, Stephen Tong, Anthea C. Lindquist

**Affiliations:** 1 Department of Obstetrics and Gynaecology, University of Melbourne, Melbourne, Victoria, Australia; 2 Mercy Perinatal, Mercy Hospital for Women, Heidelberg, Victoria, Australia; Flinders University, AUSTRALIA

## Abstract

**Objectives:**

Sex impacts birthweight, with male babies heavier on average. Birthweight charts are thus sex specific, but ultrasound fetal weights are often reported by sex neutral standards. We aimed to identify what proportion of infants would be re-classified as SGA if sex-specific charts were used, and if this had a measurable impact on perinatal outcomes.

**Methods:**

Retrospective cohort study including all infants born in Victoria, Australia, from 2005–2015 (529,261 cases). We applied GROW centiles, either adjusted or not adjusted for fetal sex. We compared overall SGA populations, and the populations of males considered small by sex-specific charts only (SGA_sex-only_), and females considered small by sex-neutral charts only (SGA_unadjust-only_).

**Results:**

Of those <10^th^ centile by sex-neutral charts, 39.6% were male and 60.5% female, but using sex-specific charts, 50.3% were male and 49.7% female. 19.2% of SGA females were reclassified as average for gestational age (AGA) using sex-specific charts. These female newborns were not at increased risk of stillbirth, combined perinatal mortality, NICU admissions, low Apgars or emergency CS compared with an AGA infant, but were at greater risk of being iatrogenically delivered on suspicion of growth restriction. 25.0% male infants were reclassified as SGA by sex-specific charts. These male newborns, compared to the AGA_all_ infant, were at greater risk of stillbirth (RR 1.94, 95%CI 1.30–2.90), combined perinatal mortality (RR 1.80, 95%CI 1.26–2.57), NICU admissions (RR 1.38, 95%CI 1.12–1.71), Apgars <7 at 5 minutes (RR 1.40, 95%CI 1.25–1.56) and emergency CS (RR 1.12, 95%CI 1.06–1.18).

**Conclusions:**

Use of growth centiles not adjusted for fetal sex disproportionately classifies female infants as SGA, increasing their risk of unnecessary intervention, and fails to identify a cohort of male infants at increased risk of adverse outcomes, including stillbirth. Sex-specific charts may help inform decisions and improve outcomes.

## Introduction

Growth restriction is the failure of the fetus to reach its full growth potential. It is associated with a higher risk of stillbirth [1–4], short term neonatal morbidity [5–10], adverse neurodevelopmental outcomes [8,11–13] and poorer long-term adult health [14,15]. Much of antenatal care centres around the detection, monitoring and timely delivery of the growth restricted fetus [16–19]. However, defining growth restriction is challenging. The optimal weight for any individual fetus, and thus its failure to achieve it, is unknown. Hence, comparisons are made to others of the same gestational age. Those that are smaller, such as being small for gestational (SGA, <10^th^ centile), become the focus for intervention. It is therefore important that the optimal growth standards are used to classify fetuses as small and at risk, or appropriately grown.

There is considerable debate regarding which growth standard is best to detect fetuses at risk of adverse outcome [20,21]. Some enthusiasts advocate that growth standards should be customised for fetal and maternal characteristics known to impact birthweight [22]. Others suggest that some or all of these factors may be on the causal pathway to placental insufficiency (and therefore no statistical adjustment should be made for them), and that a single international standard for all fetuses should be adopted [23]. However, most would agree that expected birthweight should be adjusted for factors that are truly physiological [24]. Fetal sex may be one such factor.

It is well-understood that fetal sex plays a role in determining birthweight [24–26], where females have a lower average birthweight [26,27]. Sex specific differences in growth are present across the second and third trimesters [28–30]. These differences have often been accepted as physiological, despite a lack of empirical evidence to support it. Confusion surrounding the importance, or lack thereof, of adjusting for fetal sex is highlighted by the fact that many ultrasound derived charts estimating fetal weight (used by clinicians to determine delivery decisions) do not adjust for sex [29,31], yet most charts used to interpret the actual birthweight are sex-specific [27,32].

If physiological sex-specific differences exist, using charts unadjusted for fetal sex may be underestimating the true proportion of males, and overestimating the proportion of females, experiencing placental insufficiency. Adjusting for fetal sex may represent an opportunity to improve existing growth standards, in a way that is acceptable to the broader obstetric community.

Using a large population cohort, we aimed to assess the impact of using sex-specific (rather than unadjusted) charts on the classification of infants as SGA, and whether this reclassification had a measurable impact on the association of SGA with perinatal morbidity and mortality. We hoped this would clarify whether the difference in male and female birthweights was truly physiological, thus providing support for universal adoption of sex-specific charts. Importantly, we examined whether adjusting for fetal sex may strengthen associations with severe perinatal morbidity and mortality, and thus has the potential to avoid unnecessary induction of labour.

## Materials and methods

### Population and data collection

A retrospective cohort study was conducted using data on all infants born in Victoria, Australia, between 2005 to 2015. Data were obtained from the Consultative Council on Obstetric and Paediatric Mortality and Morbidity (CCCOPMM), the central agency that collects data on obstetric and perinatal outcomes within the state [33,34].

Prior to data cleaning and analysis, an *a priori* plan was formulated to determine the inclusion and exclusion criteria and how implausible data values would be managed. Singleton pregnancies from 24+0 to 42+6 weeks’ gestation at delivery were included. Those less than 24 weeks’ gestation were excluded due to significant variation in resuscitation preferences and outcomes. Other exclusion criteria included multiple pregnancy, congenital anomalies, termination of pregnancy or those with missing or implausible birthweights or missing infant sex data. Given the importance of gestation in determining a birthweight centile, those in whom gestation in days was not recorded, or those with uncertainty regarding the exact gestation, were also excluded.

Gestation in days was calculated based on the date of birth and the last normal menstrual period (before 2009) or the date of birth and estimated due date (after 2009), which incorporated first trimester ultrasound confirmation of estimated due date if available. Maternal height and weight data were based on that recorded at the obstetric booking visit. Parity was defined as the number of previous births (live or stillborn) over 20 weeks’ gestation. Maternal age was recorded to the nearest year at booking, and birthweight was recorded in grams. Indigenous status was classified based on self-identification as an Indigenous or Torres Strait Islander Australian. Country of birth was also self-reported. Obstetric and perinatal outcomes were collected routinely by the attending midwives during pregnancy, birth, and the postnatal period.

### Definitions of SGA

We used Gestation Related Optimal Weight (GROW) charts to compare customised and uncustomised data for fetal sex. GROW charts predict optimal fetal growth at term, with a proportionality fetal weight curve, extrapolated backwards and based on Hadlock’s formula [35], at earlier gestations. Given GROW charts are derived from a fetal weight standard, they are appropriate for use at preterm as well as term gestations [36]. They provide an option for generic birthweight centiles if no fetal sex is provided, or sex-specific centiles if male and female sex data are available. Although GROW also provides the option of customisation on maternal characteristics (parity, ethnicity, maternal height and weight), we did not customise on any of these parameters, in order to assess only the impact of sex-specific standards.

In order to assess the specific effect of known fetal sex and therefore sex customisation on perinatal outcomes, the SGA population was calculated for a ‘sex-specific’ standard (SGA_sex_; customised for fetal sex), and an ‘unadjusted’ standard (SGA_unadjust_; not adjusted for fetal sex). We also assessed the non-overlapping SGA populations, ie. those classified as SGA by one chart but not another (SGA_unadjust-only_ and SGA_sex-only_). While most analyses were performed using <10^th^ centile, we additionally examined proportions of males and females classified as <3^rd^ centile by each chart, as a marker of severe growth restriction.

For statistical analyses, we defined two additional reference groups. SGA_all_ was defined as those infants classified as <10^th^ centile by both customised and uncustomised standards, and AGA_all_, which was defined as those >10^th^ centile and <90^th^ centile by both standards (appropriate for gestational age by both standards). SGA_all_ was used to identify an unequivocally high-risk cohort, and AGA_all_ a lower risk reference group.

### Outcomes

Our primary outcome was the risk of stillbirth within the <10^th^ centile cohorts discussed above. We assessed this risk in two ways.

1. We looked at the difference in relative risk of stillbirth among SGA male infants compared with SGA female infants, using a) unadjusted, and b) sex-specific charts. We hypothesised that if the differences in birthweight were physiological, then the relative risk of stillbirth would be equivalent using sex-specific charts, and unequal using unadjusted charts (which would have misclassified some females as being, and males not being, SGA).
2. We compared the relative risk in the cohort of infants identified as SGA by one chart, but not another (SGA_unadjust-only_ and SGA_sex-only_) to the AGA_all_ reference group. If sex-specific charts were superior, then the male infants classified only by sex-specific charts (SGA_sex-only_) would be higher risk than the AGA_all_ infant (ie. correctly classified as pathologically small). We also hypothesised that if sex-specific charts were better, then the female infants classified only by unadjusted charts (SGA_unadjust-only_) would be relatively low risk (ie. incorrectly classified as small).

Secondary outcomes included neonatal mortality (neonatal death within 28 days of life), combined perinatal mortality (stillbirth or neonatal death within 28 days of life), low five-minute Apgar scores of <7 or <4, admission to the Neonatal Intensive Care Unit (NICU), instrumental assisted birth and emergency caesarean section rates. ‘Suspected poor fetal growth’ as the documented reason for induction of labour, or ‘fetal distress’ as a reason for operative birth (instrumental birth or caesarean section), were additional secondary outcomes. The former was used as a measure of which infants were considered growth restricted antenatally, and the latter a measure of increased likelihood of true growth restriction, with the hypoxic challenge of labour unmasking placental insufficiency.

### Statistical analysis

Baseline characteristics of the population were summarized by mean (standard deviation), median (25^th^– 75^th^ percentile) and number (%) according to type and distribution of the data. For each SGA classification and non-overlapping population, outcomes were reported as the point estimate with Wilson 95% confidence intervals. Significance level was two-sided, set at 0.05 and not adjusted for multiple comparisons. Statistical analysis was conducted using Stata Version 16 (StataCorp. 2019. Stata Statistical Software: Release 16.1. College Station, TX, USA).

### Ethics

Ethical approval for the project was obtained from the Mercy Health Human Research Ethics Committee (approval project number R16-10). As this was a retrospective cohort study using de-identified data, individual patient consent was not required.

## Results

### Study population

**Fig 1:**
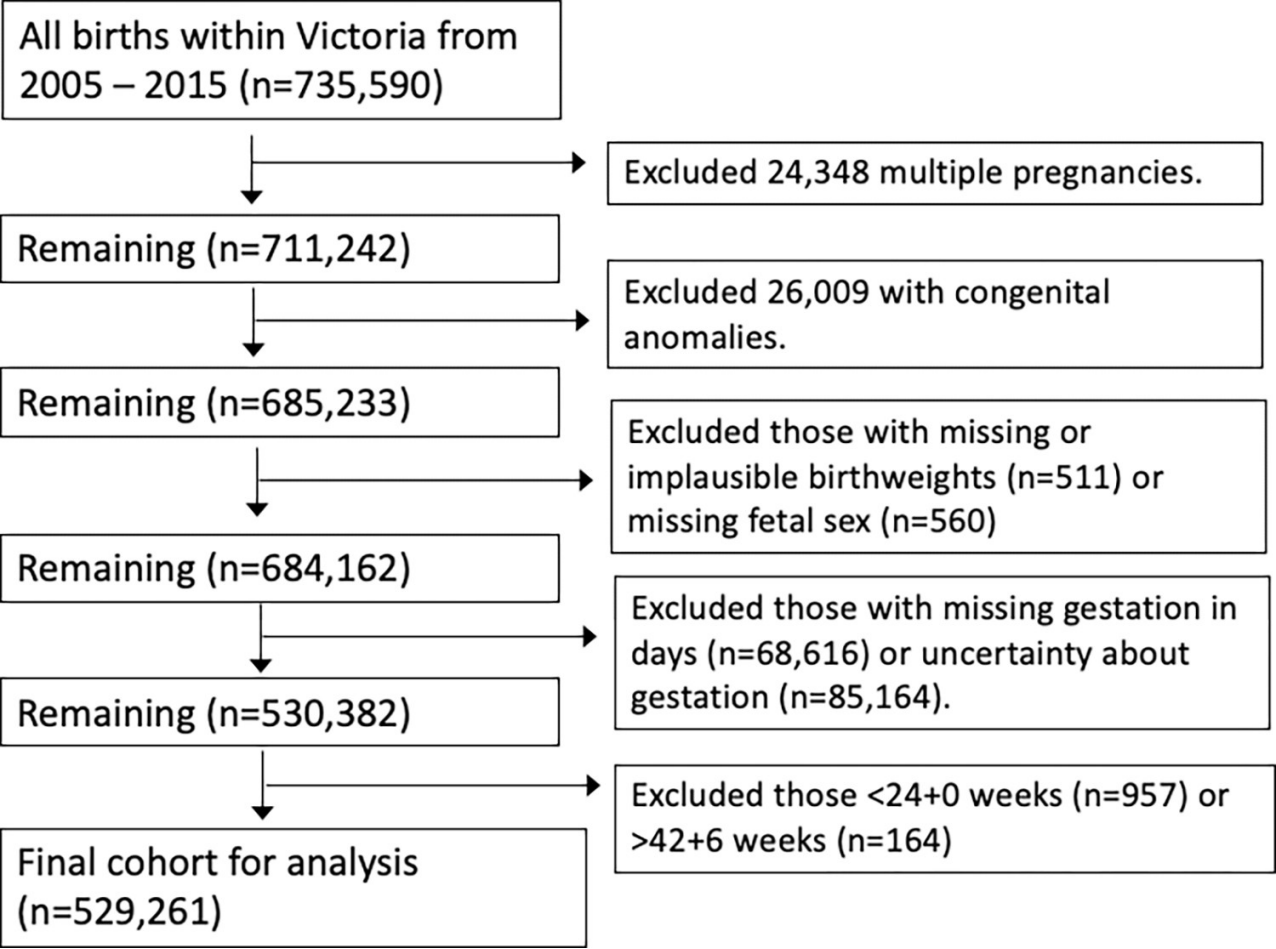
Flow diagram of exclusions.

Between 2005–2015 there were 735,591 births in Victoria. After excluding multiple pregnancies, congenital anomalies, those under 24+0 or over 42+6 weeks gestation, those with missing or uncertain gestation data or missing or implausible birthweights, there were 529,261 infants available for analysis (Fig 1). 269,541 (50.9%) were male and 259,720 (49.1%) female. Overall, there were 1,479 stillbirths, of which 759 (51.3%) were male and 720 (48.7%) female.

### Classification of SGA using adjusted and unadjusted charts

Of the 81,447 infants classified as <10^th^ centile birthweight by unadjusted charts, 32,226 (39.6%) were male, and 49,221 (60.4%) were female. Of the 80,046 infants classified as <10^th^ centile by sex-specific charts, 40,274 (50.3%) were male, and 39,772 (49.7%) were female (Table 1).

**Table 1:** Baseline characteristics for different cohorts and non-overlapping populations classified as <10^th^ centile by charts customised or uncustomised on fetal sex.

	AGA_all_ (n = 377,045)	SGA_all_ (n = 71,998)	SGA_unadjust_ (n = 81,447)	SGA_unadjust-only (females)_ (n = 9,449)	SGA_sex_ (n = 80,046)	SGA_sex-only (males)_ (n = 8,048)
Definition	Average for gestational age (10-90^th^ centile) by both charts	Small for gestational age (<10^th^ centile) by both charts	SGA by charts not customised for fetal sex	Females that are SGA only by unadjusted charts, but not when sex is adjusted for	SGA by sex-specific charts.	Males that are SGA only by sex-specific charts, but not unadjusted charts
**Male infant (%)**	193,062 (51.2)	32,226 (44.8)	32,226 (39.6)	-	40,274 (50.3)	8,048 (100)
**Female infant (%)**	183,983 (48.8)	39,772 (55.2)	49,221 (60.4)	9,449 (100)	39,772 (49.7)	-
**Birthweight** ***Mean (SD)***	3,447 (384)	2,689 (433)	2,720 (431)	2,972 (271)	2,722 (427)	3,001 (300)
**Gestational age of delivery (weeks)** ***Median IQR)***	39.7 (38.7–40.6)	39.4 (38.3–40.4)	39.6 (38.4–40.6)	39.7 (38.7–40.6)	39.6 (38.4–40.6)	39.6 (38.6–40.6)
**Maternal age** ***Mean (SD)***	31.0 (5.3)	30.2 (5.5)	30.3 (5.5)	30.4 (5.4)	30.2 (5.5)	30.4 (5.4)
**Body Mass Index** ***Mean (SD)***	25.8 (5.6)	24.6 (5.3)	24.6 (5.3)	24.8 (5.3)	24.6 (5.3)	24.7 (5.3)
**Nulliparous n (%)**	163,894 (43.5)	41,592 (57.8)	46,612 (57.2)	5,020 (53.1)	46,015 (57.5)	4,423 (55.0)
**Mother overseas born (%)**	116,224 (30.8)	28,863 (40.1)	32,366 (39.7)	3,503 (37.1)	32,009 (40.0)	3,146 (39.1)

Above: AGA_all._ = Average for gestational age (10-90^th^ centile) by both charts. SGA_all_ = Small for gestational age (<10^th^ centile) by both charts. SGA_unadjust_ = SGA by charts not customised for fetal sex. SGA_unadjust-only (females)_ = Female cohort considered SGA only by uncustomised charts, and not when fetal sex is adjusted for. SGA_sex_ = SGA by charts customised for fetal sex. SGA_sex-only (males)_ = Male cohort considered SGA only by charts customised for fetal sex.

**Fig 2:**
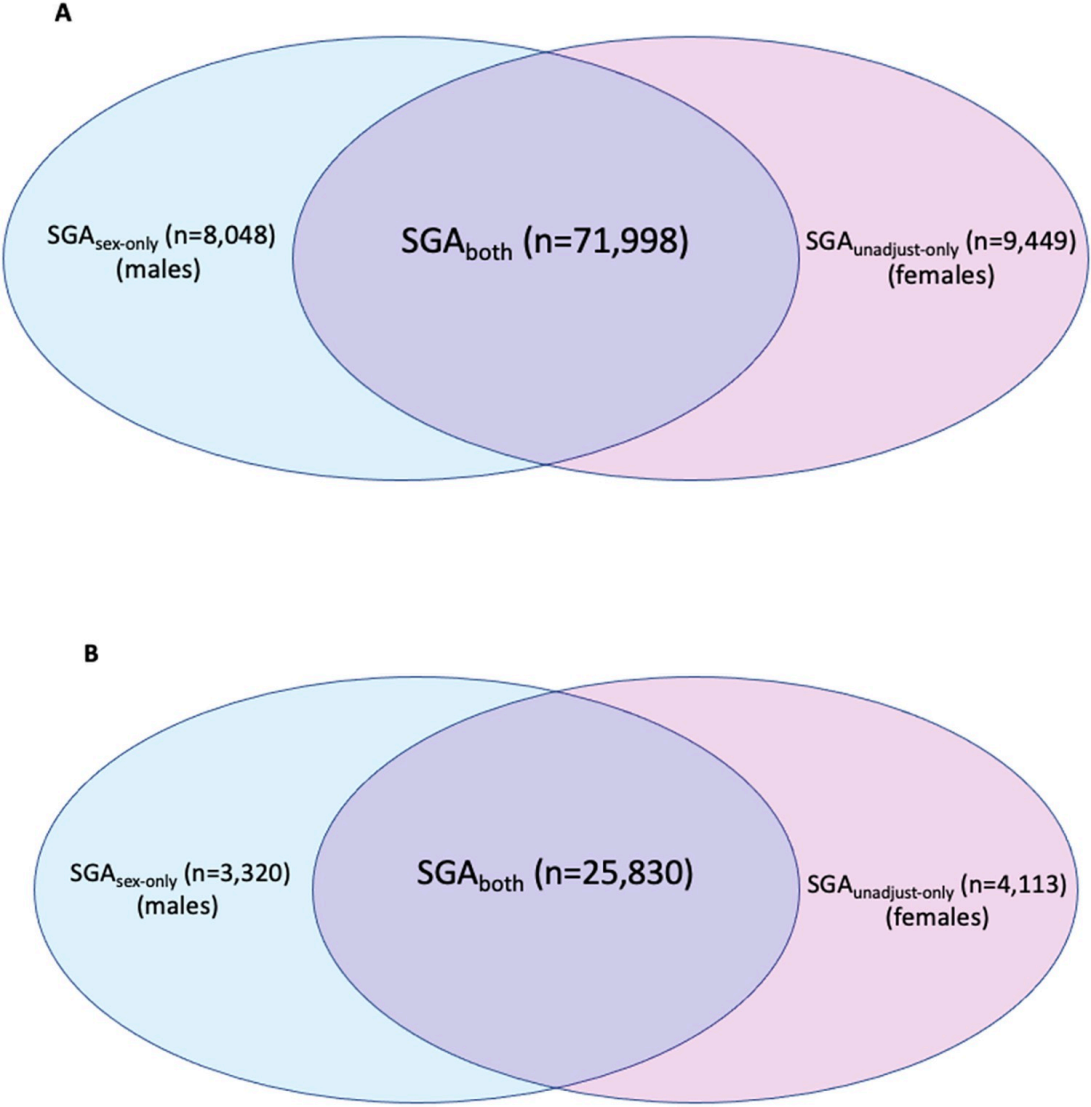
**A-B:** Venn diagram of non-overlapping populations of infants classified as A) <10^th^ centile and B) <3^rd^ centile by sex-specific or unadjusted charts.

For infants classified as <3^rd^ centile by unadjusted charts, 11,408 (38.1%) were male, and 18,535 (61.9%) female. Of those infants <3^rd^ centile by sex-specific charts, 14,728 (50.5%) were male, and 14,422 (49.5%) female (Fig 2B).

Adjusting for fetal sex meant a further 8,048 (25.0%) males were reclassified as <10^th^ centile (SGA_sex-only,_ blue) and 9,449 (19.2%) female infants were reclassified as >10^th^ centile (Fig 1A) (SGA_unadjust-only,_ pink). As a percentage of the overall population of infants, 3.6% of females and 3.0% of males were reclassified after fetal sex customisation.

For <3^rd^ centile, adjustment for fetal sex resulted in a further 3,320 of males reclassified as <3^rd^ centile (29% increase), while 4,113 fewer females were classified as <3^rd^ centile (22.2% reduction) (Fig 1B). As a percentage of the overall population of infants, this reclassified 1.2% of males and 1.6% of females.

Comparison between the non-overlapping groups therefore identified two new sub-populations of interest: 1) SGA_sex-only_, which consisted of male infants that were considered AGA by unadjusted charts, but SGA when customised on sex (ie centile adjusted by higher expected weight); and 2) SGA_unadjust-only,_ which consisted of female infants considered SGA by unadjusted charts, but AGA when customised (ie centile adjusted by lower expected weight).

For the <10^th^ centile SGA subgroups and non-overlapping populations, baseline maternal and obstetric characteristics were compared (Table 1).

### Stillbirth rates for male and females using unadjusted and sex-specific charts

When unadjusted charts were used, the relative stillbirth rate was higher within the male SGA cohort (11.0 per 1000) compared with the female SGA cohort (7.8 per 1000, p<0.0001) (Table 2). When sex-specific charts were used, the stillbirth rates were equivalent between males and females (9.4 per 1000 for male, 9.3 per 1000 for females, p = 0.788).

**Table 2:** Number of stillborn infants, stillborn rates, and relative risk of stillbirth, out of the male and female SGA cohorts as classified by unadjusted and sex-specific charts, at preterm and term gestations.

	UNADJUSTED CHARTS		SEX-SPECIFIC CHARTS	
	Male	Female	Male	Female
**ALL GESTATIONS (N = 529,261)**				
Total number by sex	269,541 (50.9)	259,720 (49.1)	269,541 (50.9)	259,720 (49.1)
SGA number (% of sex-specific population)	32,226 (12.0)	49,221 (19.0)	40,274 (14.9)	39,772 (15.3)
Stillbirths classified as SGA (n) Rate/1000 births* Relative risk (95% CI)	355 11.0 *Ref*	385 7.8 *0*.*71 (0*.*62–0*.*82)*, *p<0*.*0001*	380 9.4 *Ref*	368 9.3 *0*.*98 (0*.*85–1*.*13)*, *p = 0*.*788*
**PRETERM ONLY (<37 WEEKS, N = 28,963)**				
Total number by sex	15,783 (54.5)	13,180 (45.5)	15,783 (54.5)	13,180 (45.5)
SGA number (% of sex-specific preterm population)	3,659 (23.2)	4,305 (32.7)	4,191 (26.6)	3,840 (29.1)
Stillbirths classified as SGA (n) Rate/1000 births* Relative risk (95% CI)	265 72.4 *Ref*	269 62.5 *0*.*86 (0*.*73–1*.*02)*, *p = 0*.*078*	278 66.3 *Ref*	259 67.4 *1*.*02 (0*.*86–1*.*20)*, *p = 0*.*842*
**TERM ONLY (> = 37 WEEKS, N = 500,298)**				
Total number by sex	253,758	246,540	253,758	246,540
SGA number (% of sex-specific term population)	28,567 (11.3)	44,916 (18.2)	36,083 (14.2)	35,932 (14.6)
Stillbirths classified as SGA (n) Rate/1000 births* Relative risk (95% CI)	90 3.2 *Ref*	116 2.6 *0*.*82 (0*.*62–1*.*08)*, *p = 0*.*156*	102 2.8 *Ref*	109 3.0 *1*.*07 (0*.*82–1*.*41)*, *p = 0*.*608*

*Rate/1000 births calculated as number of SGA stillbirths divided by total number of births considered SGA by that classification.

### Analysis of all obstetric and perinatal outcomes

Table 3 details the obstetric and perinatal outcomes for SGA cohorts and non-overlapping populations. As expected, all adverse outcomes, including stillbirth, were significantly more likely across all SGA groups (SGA_all,_ SGA_unadjust,_ SGA_sex_) than in the AGA_all_ (reference) group.

**Table 3:** Obstetric and perinatal outcomes for non-overlapping populations classified as <10^th^ centile by charts customised or uncustomised on fetal sex.

	AGA_all_ (n = 377045)	SGA_all_ (n = 71998)	SGA_unadjust_ (n = 81447)	SGA_unadjust-only (females)_ (n = 9449)	SGA_sex_ (n = 80046)	SGA_sex-only (males)_ (n = 8048)
	Average for gestational age (10-90^th^ centile) by both charts	Small for gestational age (<10^th^ centile) by both charts	SGA by charts not customised for fetal sex	Females that are SGA only by unadjusted charts, but not when sex is adjusted for	SGA by sex-specific charts.	Males that are SGA only by sex-specific charts, but not unadjusted charts
**Stillbirth n (%)** **RR (95% CI)**	603 (0.2) *Ref*	723 (1.0) *6*.*28 (5*.*64–6*.*99*, *p<0*.*0001)*	740 (0.9) *5*.*68 (5*.*10–6*.*32*, *p<0*.*0001)*	17 (0.2) *1*.*12 (0*.*70–1*.*82*, *p = 0*.*63)*	748 (0.93) *5*.*84 (5*.*25–6*.*50*, *p<0*.*0001)*	25 (0.3) *1*.*94 (1*.*30–2*.*90)*, *p = 0*.*0009*
**Combined perinatal mortality n (%)** **RR (95% CI)**	807 (0.2) *Ref*	797 (1.1) *5*.*17 (4*.*69–5*.*71*, *p<0*.*0001)*	824 (1.01) *4*.*73 (4*.*29–5*.*21*, *p<0*.*0001)*	27 (0.3) *1*.*34 (0*.*91–1*.*96*, *p = 0*.*14)*	828 (1.0) *4*.*83 (4*.*39–5*.*32*, *p<0*.*0001)*	31 (0.4) *1*.*80 (1*.*26–2*.*57*, *p-0*.*001)*
**Admission to NICU n(%)** **RR (95% CI)**	2879 (0.76) *Ref*	1529 (2.12) *2*.*78 (2*.*61–2*.*96*, *p<0*.*0001)*	1612 (2.0) *2*.*59 (2*.*44–2*.*75*, *p<0*.*0001)*	83 (0.9) *1*.*15 (0*.*93–1*.*43*, *p = 0*.*21)*	1614 (2.0) *2*.*61 (2*.*46–2*.*77*, *p<0*.*0001)*	85 (1.1) *1*.*38 (1*.*12–1*.*71*, *p = 0*.*003)*
**Apgars <4 at 5 minutes (%)** **RR (95% CI)**	1539 (0.41) *Ref*	974 (1.4) *3*.*32 (3*.*07–3*.*60*, *p<0*.*0001)*	1021 (1.3) *3*.*04 (2*.*81–3*.*29*, *p<0*.*0001)*	47 (0.5) *1*.*22 (0*.*91–1*.*63*, *p = 0*.*18)*	1017 (1.3) *3*.*12 (2*.*89–3*.*37*, *p<0*.*0001)*	43 (0.5) *1*.*31 (0*.*97–1*.*77*, *p = 0*.*08)*
**Apgars <7 at 5 minutes (%)** **RR (95% CI)**	10248 (2.7) *Ref*	3552 (5.0) *1*.*82 (1*.*75–1*.*89*, *p<0*.*0001)*	3817 (4.7) *1*.*73 (1*.*67–1*.*79*, *p<0*.*0001)*	265 (2.8) *1*.*03 (0*.*92–1*.*16*, *p = 0*.*60)*	3857 (4.8) *1*.*78 (1*.*71–1*.*84*, *p<0*.*0001)*	305 (3.8) *1*.*40 (1*.*25–1*.*56*, *p<0*.*0001)*
**Suspected poor growth indication for induction (%)** **RR (95% CI)**	2741 (0.73) *Ref*	7562 (10.5) *14*.*5 (13*.*8–15*.*1*, *p<0*.*0001)*	7899 (9.7) *13*.*3 (12*.*8–13*.*9*, *p<0*.*0001)*	337 (3.6) *4*.*90 (4*.*39–5*.*48*, *p<0*.*0001)*	7819 (9.8) *13*.*4 (12*.*9–14*.*0*, *p<0*.*0001)*	248 (3.1) *4*.*3 (3*.*73–4*.*81*, *p<0*.*0001*
**Suspected stress in labour indication for operative birth (%)** **RR (95% CI)**	40382 (10.7) *Ref*	13419 (18.6) *1*.*74 (1*.*71–1*.*77*, *p<0*.*0001)*	14718 (18.1) *1*.*69 (1*.*66–1*.*72*, *p<0*.*0001)*	1299 (13.8) *1*.*28 (1*.*22–1*.*35*, *p<0*.*0001)*	14738 (18.4) *1*.*72 (1*.*69–1*.*75*, *p<0*.*0001)*	1319 (16.4) *1*.*53 (1*.*46–1*.*61*, *p<0*.*0001)*
**Emergency caesarean section (%)** **RR (95% CI)**	51759 (13.7) *Ref*	13108 (18.2) *1*.*33 (1*.*30–1*.*35*, *p<0*.*0001)*	14306 (17.6) *1*.*28 (1*.*26–1*.*30*, *p<0*.*0001)*	1198 (12.7) *0*.*94 (0*.*88–0*.*97*, *p = 0*.*003)*	14347 (17.9) *1*.*31 (1*.*28–1*.*33*, *p<0*.*0001)*	1239 (15.4) *1*.*12 (1*.*06–1*.*18*, *p<0*.*0001)*

Above: AGA_all._ = Average for gestational age (10-90^th^ centile) by both charts. SGA_all_ = Small for gestational age (<10^th^ centile) by both charts. SGA_unadjust_ = SGA by charts not customised for fetal sex. SGA_unadjust-only (females)_ = Female cohort considered SGA only by uncustomised charts, and not when fetal sex is adjusted for. SGA_sex_ = SGA by charts customised for fetal sex. SGA_sex-only (males)_ = Male cohort considered SGA only by charts customised for fetal sex. Combined perinatal mortality = combination of stillbirths and neonatal deaths <28 days of life.

**Fig 3:**
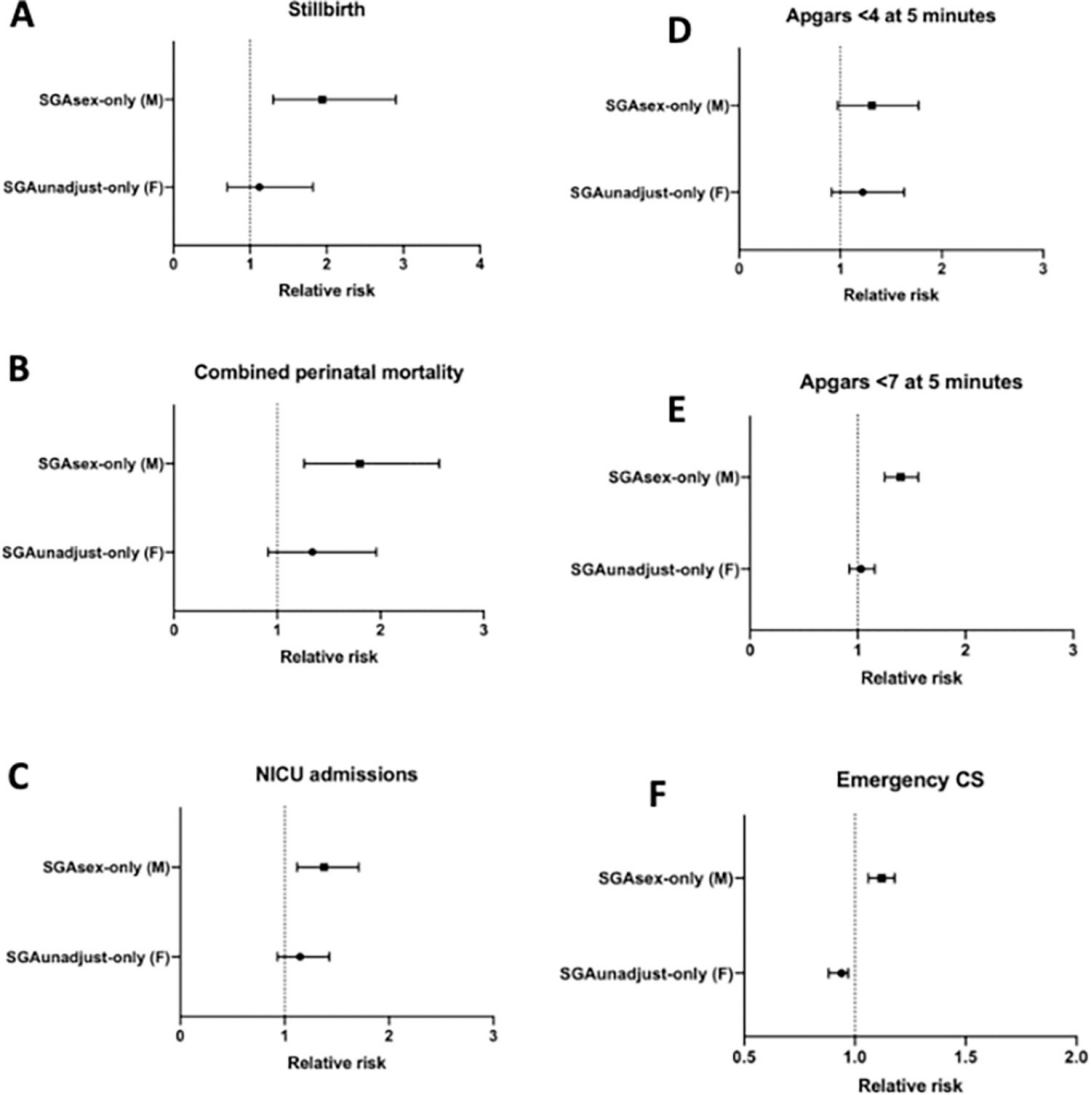
**A-F.** Comparison of relative risk ratios for the SGA population captured only by unadjusted charts (SGA_unadjust-only_, females), and those captured only by sex-specific charts (SGA_sex-only_, males) with the AGA _all_ population for A) Stillbirth, B) Combined perinatal mortality, C) NICU admissions, D) Apgars <4 at 5 minutes, E) Apgars <7 at 5 minutes and F) Emergency caesarean section rates.

We then compared the two non-overlapping populations to the AGA_all_ group. The cohort of females considered SGA only by unadjusted charts (SGA_unadjust-only_; classified as AGA using sex-specific charts) were not at significantly increased risk of stillbirth, combined perinatal mortality, NICU admission, or low Apgar score at 5 minutes. However, compared with the AGA_all_ group, they were significantly more likely to have had a planned delivery as a result of suspected fetal growth restriction (RR 4.90, 95% CI 4.39–5.48), and to have had suspected fetal distress in labour as an indication for operative birth (RR 1.28, 95% CI 1.22–1.35). They were significantly less likely to have ended up with an emergency caesarean section (Table 3, Fig 3A–3F).

The cohort of males considered SGA by sex-specific charts only (SGA_sex-only;_ classified as AGA using unadjusted charts) were at significantly increased risk of nearly all the adverse outcomes we examined, compared with the AGA_all_ infant. These include stillbirth (RR 1.94, 95% CI 1.30–2.90), combined perinatal mortality (RR 1.80, 95% CI 1.80 (1.26–2.57), NICU admission (RR 1.38, 95% CI 1.12–1.71), Apgar score <7 at 5 minutes (RR 1.40, 95% CI 1.25–1.56) and emergency caesarean section (RR 1.12, 95% CI 1.06–1.18) (Table 2, Fig 2A–2F).

## Results

### Main findings

In a large population-based cohort study, we assessed the impact of adjusting growth standards for fetal sex on perinatal morbidity and mortality amongst infants classified as SGA. The use of sex-specific charts identified an additional cohort of male infants who, when compared with an AGA reference group, were at increased risk of stillbirth, perinatal mortality, NICU admission, Apgar <7 at 5 minutes and emergency caesarean section. These infants would be missed if growth standards unadjusted for fetal sex were used.

The use of sex-specific charts also excluded a cohort of female infants considered <10^th^ centile by unadjusted charts. The risk of stillbirth, total perinatal mortality, NICU admission and low Apgar score were not significantly higher in these infants than the AGA_all_ cohort. However, these females were significantly more likely to have been induced for suspected poor growth than an AGA_all_ infant. This suggests that this is a cohort of female infants with low risk of adverse outcomes, but high rates of obstetric intervention.

Using sex-specific charts, which accounted for the expected higher birthweight of a male infant, the proportion of male infants classified as SGA increased by 25%. This meant 3% of the total male population was reclassified. In contrast, the use of sex-specific charts accounted for the expected lower birthweight of a female infant and reclassified almost 20% of female newborns originally <10^th^ centile by unadjusted charts as AGA. This meant 3.6% of the entire female cohort was reclassified. We found a similar magnitude of reclassification amongst infants with a birthweight <3^rd^ centile. This centile cut-off is of particular clinical significance as it is widely applied as a surrogate measure of true placental insufficiency [37].

Our findings provide strong epidemiological evidence that a physiological difference in birthweight between male and female infants exists. Growth standards unadjusted for fetal sex classify too many females as SGA; this artificially reduces the relative risk of stillbirth amongst small females, by diluting the group with healthy female infants. Growth standards unadjusted for fetal sex classify too few males as SGA; this artificially increases the relative risk of stillbirth amongst males. When sex-specific charts are used, they classify equivalent proportions of male and female infants as small, with equivalent stillbirth risks.

Although in some situations, sex-specific growth standards are already standard practice, unadjusted charts remain widely used. Of note, many ultrasound standards are unadjusted for fetal sex, as the sex is commonly concealed at the parents’ request. This leads to the paradoxical situation that interventions are proposed using one method of interpreting estimated fetal weight (unadjusted for fetal sex), and another used to interpret the final birthweight (sex-specific). Given that estimated fetal weight centile is commonly used as a trigger for both antenatal surveillance and timing of delivery [38,39], it may be preferable that the sex is noted (but not disclosed) antenatally so that a sex-specific centile can be calculated (although this ideally needs to be demonstrated in a further study where outcomes are prospectively assessed after an ultrasound estimated fetal weight is generated). If it were proven that adjusting for sex improves precision of identifying fetuses that are small and at increased risk of perinatal morbidity, it should be feasible to take gender into account at the ultrasound while keeping this information from parents who do not wish to know until the birth.

### Interpretation

Females have lower birthweights compared with males [27]. Prescriptive birthweight standards from the WHO and INTERGROWTH-21^st^ studies are sex-specific [40,41]. Sex-specific differences in estimated fetal size have also been demonstrated in in-utero ultrasound studies from as early as 15 weeks [28,42,43], with males consistently heavier than females. While these studies have simply reported sex-specific differences, our study confirms that these differences are indeed physiological.

That the relative smallness of female fetuses is physiological, rather than pathological, is supported by reports of pathological placental lesions [44] and perinatal outcomes related to placental insufficiency [45–48] being less common in female infants. A population registry study from the Netherlands also reported a slightly increased risk of mortality amongst SGA males compared with SGA females after 28 weeks [49]. That this increase in risk was identified even when using sex-specific population charts underscores the importance of using the most precise method of identifying high risk males that are growing poorly.

The WHO study quantified the effect of fetal sex on birthweight at 3.5–4.5%. In their study, this justified designing sex-specific charts, as a difference of that magnitude would have a substantial impact on the classification of small or large for gestational age [24] In agreement with these findings, we also found that 3.3% of infants overall were reclassified. Although this absolute difference appears small, it amounts to reclassifying approximately 21.5% of the original SGA population. This would have substantial implications at a population level [50].

When exploring the performance of different growth standards to predict perinatal outcome, two vastly different methodologies are often compared [51]. These include comparisons of a fetal and birthweight chart [52], or uncustomised charts, with charts customised on multiple maternal and fetal characteristics [53]. This makes it difficult to elucidate the exact impact of a particular characteristic. Accordingly, which aspect of the growth standard led to improved correlation with adverse outcomes remains obscured. By comparing subgroups utilising the same growth standard, with only one aspect (fetal sex) adjusted, it has allowed us to clarify the exact impact that adjusting for fetal sex has on the correlation with clinical outcomes. We suggest that the more controversial aspects of growth standards, such as customising for maternal factors, can be approached in a similar way, to determine which characteristics improve association with significant adverse perinatal outcomes.

### Strengths and limitations

Our large, population cohort provides strong evidence of benefit in the use of sex-specific charts, examining highly clinically relevant outcomes, including stillbirth. Although the relative risk differences were modest, we believe that this simple change may be of benefit on a population level, and provides a case for incorporating the use of sex-specific charts in clinical practice. However, we are limited by the retrospective nature of our study. Additionally, we applied growth standards to a population of infants already born. This means that we can only hypothesise as to the potential benefit of transitioning to sex-specific growth standards on antenatal management decisions dictated by ultrasound estimated fetal weights.

## Conclusions

Our study provides strong epidemiological evidence that using sex-specific charts improves correlation with perinatal morbidity and mortality, compared with growth standards unadjusted for fetal sex. They reclassify as SGA a proportion of male infants at high risk of adverse outcomes, including stillbirth. Offsetting this is a reduction in the number of female fetuses classified as SGA, whose risk is no greater than AGA infants. It is plausible that this small, cost-neutral adjustment may reduce both false positive and false negative rates in the detection of fetal growth restriction, which could translate to meaningful improvements in obstetric and perinatal outcomes.
